# Is *Mycobacterium tuberculosis* infection life long?

**DOI:** 10.1136/bmj.l5770

**Published:** 2019-10-24

**Authors:** Marcel A Behr, Paul H Edelstein, Lalita Ramakrishnan

**Affiliations:** 1 Department of Medicine, McGill University, McGill International TB Centre, Montreal, H4A 3J1, Canada; 2 Department of Pathology and Laboratory Medicine, Perelman School of Medicine, University of Pennsylvania, Philadelphia, PA, 19104, USA; 3 Molecular Immunity Unit, Department of Medicine, University of Cambridge, MRC Laboratory of Molecular Biology, Cambridge CB2 0QH, UK

## Abstract

People with immunoreactivity to tuberculosis are thought to have lifelong asymptomatic infection and remain at risk for active tuberculosis. **Marcel A Behr and colleagues** argue that most of these people are no longer infected

**Key messages**

Two billion people worldwide are thought to be asymptomatically (latently) infected with *Mycobacterium tuberculosis* and at risk of developing active tuberculosis (TB)
The prevalence of latent TB infection is inferred from tests that detect immunoreactivity to mycobacterial antigens rather than live bacteria and from mathematical modelling
Longitudinal studies and clinical trials show that this TB immunoreactivity can persist after curative treatment
Most people with TB immunoreactivity do not develop active TB upon immunosuppression, suggesting that they have cleared their infection while retaining immunological memory to it
TB immunoreactivity cannot distinguish cleared from persistent infection, emphasising the urgent need for tests that can identify people with asymptomatic infections

A critical component of the World Health Organization’s End TB strategy is the prevention of active tuberculosis (TB) by treating people with latent TB infection.^1^ ^2^ This requires knowledge of who is latently or asymptomatically infected with *Mycobacterium tuberculosis*. As there are no direct tests for asymptomatic infection, it is inferred from the presence of TB immunoreactivity, determined by either a tuberculin skin test (TST) or an interferon gamma release assay (IGRA). The use of immunoreactivity as a proxy for infection is based on the assumption that those testing positive harbour live bacteria, which might be quiescent now but can spring to activity to cause disease later, especially if the host becomes immunocompromised.^2^ ^3^

Is this assumption valid? Knowing this would be useful for several interested parties—public health officials, TB researchers, people showing TB immunoreactivity, and doctors. We tested the assumption that TB immunoreactivity equals persistent infection by analysing the natural history of TB immunoreactivity in people given preventive treatment and of active TB in immunoreactive people with various forms of severe immunosuppression.

## Immunoreactivity does not reflect continued infection

**Fig 1:**
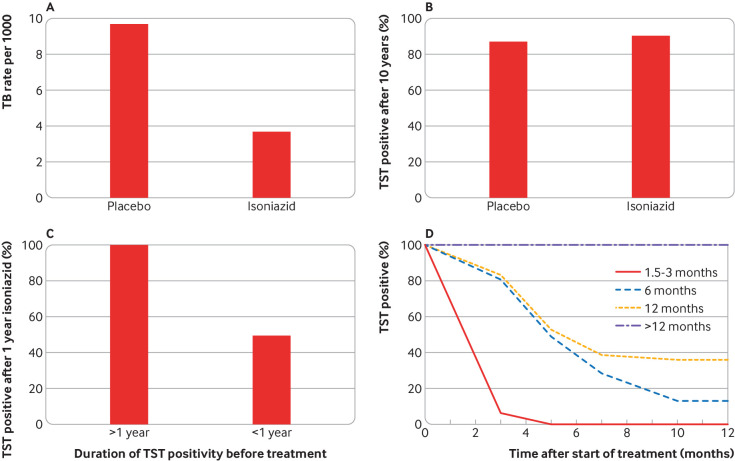
TB immunoreactivity reversion over time. A) Efficacy of one year of isoniazid prophylaxis (n=9382) versus placebo (n=9140) over a 10 year observation period, including the year of treatment, for residents of US mental institutions.^4^ Isoniazid treatment reduced TB disease by 62% over the observation period. B) TB immunoreactivity over a 10 year observation period for residents of Milledgeville mental institution participating in a placebo controlled (n=697) trial of isoniazid treatment (n=686) to prevent TB infection, all of whom were TB immunoreactive at enrolment.^4^ C) TB immunoreactivity for University of Virginia Hospital workers with known duration of TST positivity (<1 year (n=20) and >1 year (n=17)) before isoniazid treatment for one year.^5^ D) TB immunoreactivity for US navy sailors with known duration of TST positivity (1.5-3 months (n=45), 6 months (n=98), 12 months (n=36), and >12 months (n=24)) before isoniazid treatment for one year.^6^ TST=tuberculin skin test.

For TB immunoreactivity to be a reliable proxy for persistent TB infection, it must track faithfully with infection. This means that people whose infection is cured should become non-reactive. Multiple studies show that this is not the case. The large US isoniazid chemoprophylaxis trial conducted between 1956 and 1966 showed that treatment of people with TB immunoreactivity (TST results ≥10 mm) for one year lowered the incidence of active TB by 60-70% over the next nine years (fig 1A).^4^ Yet the treated people remained positive on TST up to nine years after treatment (fig 1B), showing that TB immunoreactivity can outlast elimination of infection by at least nine years.

This finding was validated by two studies that provided mechanistic insights. Among hospital employees, Atuk and Hunt showed that patients who had positive results on TSTs for more than a year before treatment remained so after treatment, whereas 50% of those who had been positive for less than a year became negative or had smaller TST induration size (fig 1C).^5^ Houk and colleagues’ study of US navy sailors treated with isoniazid for TB infection provided more granularity.^6^ All who were immunoreactive for more than a year before treatment remained so after treatment; among the remainder, those who were immunoreactive the longest were most likely to remain so after isoniazid treatment (fig 1D). These findings are consistent with the development of a lasting immunological memory that becomes more robust with longer exposure to antigen. Finally, the finding that TB immunoreactivity outlasts infection holds in the context of active TB—people remain immunoreactive after completing treatment with highly effective multidrug regimens.^7^ ^8^

So TB immunoreactivity is not a marker for the presence of continued TB infection. Rather it serves as a sign of having been infected with TB but does not reflect the outcome—bacterial clearance versus persistence.

## Immunosuppression and risk of TB

Immunosuppression—through HIV infection or medical intervention (tumour necrosis factor (TNF) inhibition, solid organ transplantation, and haematopoietic stem cell transplantation)—is associated with an increased risk of active TB in multiple studies. We used these studies to infer the frequency of persistent infection in people showing TB immunoreactivity (see supplementary table 1 and supplementary methods for details on data sources and analysis).

### TNF inhibition

**Fig 2:**
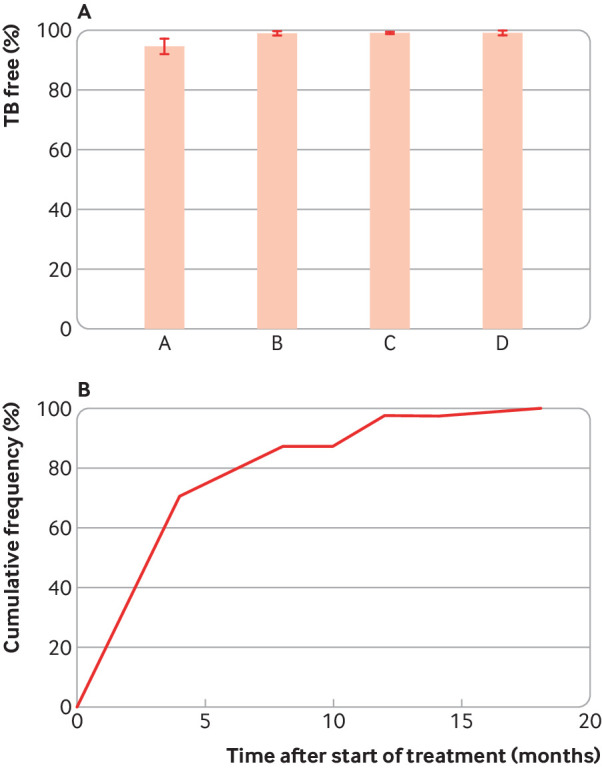
Percentage of likely or known TB immunoreactive patients who developed TB after infliximab treatment and the kinetics of TB development in these patients. A) Percentage of patients receiving infliximab who were free of TB after infliximab treatment, where columns A, B, C, and D are based on data from Gomez-Reino et al (n=1578),^13^ Baldin et al (n=1154),^12^ Keane et al (n=2931),^11^ and Wolfe et al (n=6460).^14^ B) The cumulative frequency of TB development in infliximab treated patients versus the time to develop TB after starting infliximab (n=76) from Keane et al, Gomez-Reino et al, and Wolfe et al.^11^ ^13^ ^14^ The median, average, and 75th centile time to develop TB after initiation of infliximab were 3, 4.5, and 5.6 months, respectively. Error bars indicate 95% confidence intervals.

TNF is known to be a potent determinant of resistance to TB in animal disease models.^9^ ^10^ The increased risk of TB in humans receiving the anti-TNF antibody infliximab was shown first in 2001 in a large US patient cohort^11^ and then in three subsequent studies in the US, France, and Spain (supplementary table 1 and supplementary methods).^12^ ^13^ ^14^ In these studies, performed before isoniazid prophylaxis was routinely given to TB immunoreactive patients, 0.6-4.9% of people with positive TSTs developed TB; the rest remained free of TB over the next 1.5 to 3 years (fig 2A).^11^ ^12^ ^13^ ^14^ Because the majority of cases occurred early after starting anti-TNF treatment (median time of three months) (fig 2B), TB probably resulted from pre-existing remote infection rather than from newly acquired infection. Together, these analyses show that >95% of people with positive TSTs do not have quiescent bacteria that can be roused by TNF suppression.

### HIV

**Fig 3:**
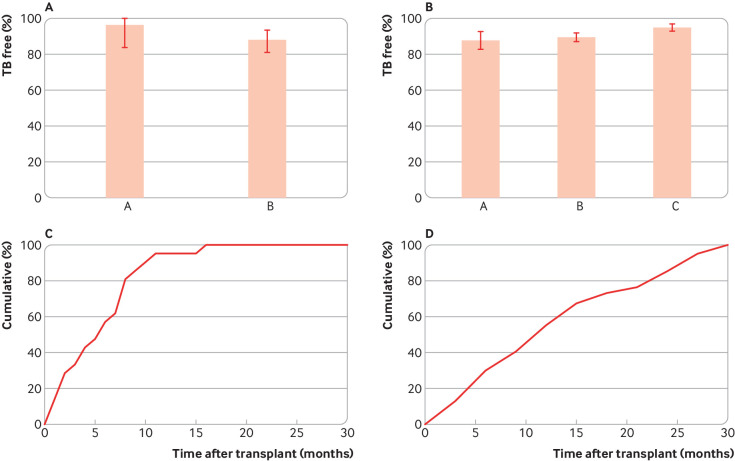
Percentage of patients with likely or known TB immunoreactivity who did not develop TB with immunosuppression caused by HIV/AIDS or solid organ transplantation. A) Patients with HIV/AIDS. Data from Moss et al (A) (n=35)^15^ and Horsburgh et al (B) (n=106),^16^ which used molecular methods to distinguish disease probably due to remote versus new infection. Only disease due to remote TB infection is shown, in those who had not received full courses of isoniazid preventive therapy. The observation periods were 2-5 years. B) After solid organ transplantation. Columns A, B, and C show data from Atasever et al (n=443),^17^ Klote et al (n=15 870),^18^ and Torre-Cisneros et al (n=4388).^19^ C) Kinetics of TB development after solid organ transplantation (n=21).^19^ The median, mean, and 75th centile times were 6.0, 5.8, and 8.0 months after transplant, respectively. D) kinetics of TB development after renal transplantation (n=66).^18^ The median, mean, and 75th centile times were 12.0, 13.6 and 21 months after transplant, respectively. Error bars indicate 95% confidence intervals.

Cell mediated immunity, especially that mediated by CD4+ T lymphocytes, is another critical defence against TB, as shown by the increased rates of TB in people with HIV. Two studies on TB in patients with HIV were done at a time when preventive TB treatment was not routinely given to patients with HIV who were TB immunoreactive. Both studies used bacterial genotyping to distinguish newly acquired from pre-existing remote TB infection, showing that 88.7% and 97.5% of people with positive TSTs remained free of TB from their remote infection over a five year period (fig 3A).^15^ ^16^

### Solid organ transplantation

Long term medical suppression of cell mediated immunity is the cornerstone of preventing rejection after allogeneic organ transplantation. Three studies of solid organ transplant recipients who did not receive isoniazid prophylaxis were done in Spain, Turkey, and the US. Most of these patients remained free of TB (88.7-95.6%) over observation periods of up to 2.5 years (fig 3B).^17^ ^18^ ^19^ The maximum reported rate of TB in people with positive TSTs was 11.3%. TB occurred later in the transplant population than in the population receiving TNF inhibition, with a median of six months for a cohort comprising mostly non-renal transplants (67%) and 12 months for renal transplants (from a different study of exclusively renal transplants) (figs 3C and 3D). This could reflect a later temporal role for cell mediated immunity in the control of TB. The shorter time to TB in non-renal transplant recipients might reflect the higher degree of immunosuppression used. Some of the later cases might have been from newly acquired infection after the transplant, in which case the estimated risk attributable to remote infection would be even lower.

### Haematopoietic stem cell transplantation

**Fig 4:**
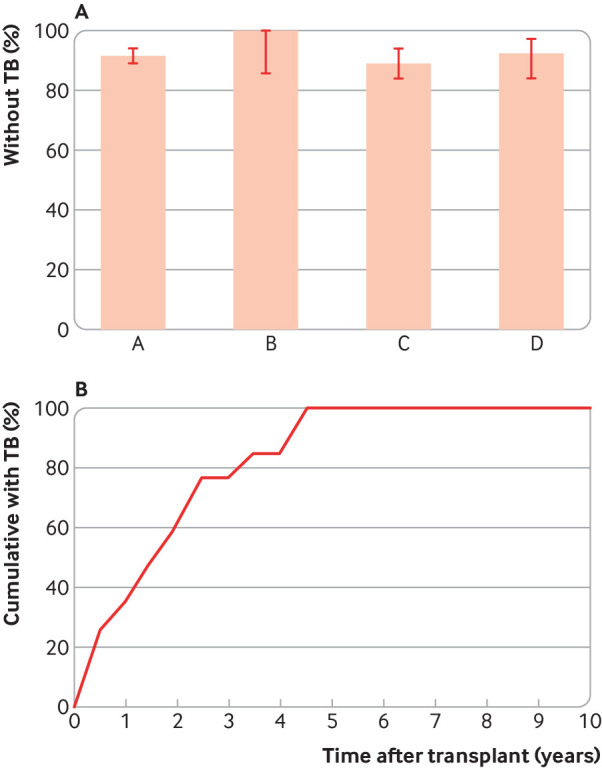
TB in patients undergoing haematopoietic stem cell transplantation. A) Proportion of people with likely TB reactivity who did not develop TB after haematopoietic stem cell transplantation. Columns A, B, C, and D data are taken from Fan et al (n=240),^20^ Cheng et al (n=29),^21^ Lee et al (n=550),^22^ and Agarwal et al (n=175),^23^ respectively. B) kinetics of TB development in 39 patients who developed TB over the 10 year observation period.^20^ The median, average, and 75th centile incubation times are 1.6, 1.8, and 2.4 years, respectively. Redrawn from Fan et al. Error bars indicate 95% confidence intervals.

Haematopoietic stem cell transplantation results in the most profound and rapid immunosuppression affecting both innate and adaptive arms of immunity; this is the most likely form of immunosuppression to cause any quiescent *M tuberculosis* to become active. We identified four cohorts of TB immunoreactive people in India, South Korea, Taiwan, and the US who underwent haematopoietic stem cell transplantation without receiving isoniazid prophylaxis.^20^ ^21^ ^22^ ^23^ The proportion of these cohorts that developed TB was less than 10% (fig 4A). In the US cohort, none of 29 participants with TB immunoreactivity developed TB after a total of 89 person years of observation. The three other studies were performed in countries with intermediate to high TB burden, yet only 7-10% of people developed TB. The median times to TB development after transplantation were 258, 368, and 445 days for the Indian, South Korean, and Taiwanese studies (fig 4B), respectively, indicating that some of these cases might have been due to new infection rather than activation of their remote infection.

## BCG and false positives

BCG vaccination can result in false positive results on TSTs in those vaccinated when older than 1 year of age.^24^ This could have falsely inflated the number of people estimated to be TB immunoreactive in the studies that we analysed. If so, the proportion of TB immunoreactive people who developed TB associated with immunosuppression would have been higher than our estimates. To ensure that this was not the case, for each study we determined the use of BCG vaccine in the appropriate country and whether the more specific IGRA tests^25^ were used to determine TB immunoreactivity. We concluded that our estimates of TB immunoreactivity were not falsely high because of BCG vaccination (see supplementary table 2).

## Summary and implications

Whether infected people can clear *M tuberculosis* has consumed TB researchers for over a century, yet multiple approaches have failed to yield a definitive answer. Our analysis of epidemiological experiments suggests the answer: between 1% and 11% of people with TB immunoreactivity continue to harbour viable bacteria capable of causing disease. In the remainder, the organisms are either dead or have lost their pathogenic potential. The latter scenario would be extraordinary. It would differ from a multitude of other quiescent infections where immunosuppression is recognised to cause a reactivation or exacerbation of infection.^26^ Cytomegalovirus, herpes simplex virus, herpes zoster virus, the protozoan *Toxoplasma gondii,* and the fungus *Cryptococcus neoformans* are known to produce full blown devastating infections from a long term latent infection.

This analysis highlights that the currently available tests for latent TB infection detect only TB reactivity—that is, immunological memory and recall responses. So they would not be expected to distinguish between long lived memory that persists after elimination of *M tuberculosis* antigens and T cell responses maintained by repeated antigen stimulation from chronic infection. Many infectious diseases (including hepatitis A, B, and C) as well as immunisation with attenuated or subunit vaccines generate memory responses (antibody, T cell, or both) that do not reflect pathogen persistence.

We hope that our analysis will stimulate further discussion about the research and biological paradigms of TB. If infection is forever, researchers will aim to study host tolerance. If infection can be cleared, researchers will try to understand the mechanisms of pathogen elimination.

Our findings indicate that the tests used in biomarker and immunological studies of persistent infection (TST and IGRA)^27^ ^28^ might have two major confounders: the control participants who do not develop TB might be a heterogeneous mix of currently and previously infected people; and efforts to find a profile that predicts increased risk of disease might be confounded by the control group (TST positive) being at reduced risk of TB, as has been shown in multiple observational studies.^29^

Notwithstanding the challenges of evaluating new biomarkers, the importance of identifying the proportion of people with persistent infection and distinguishing them from those with immunological memory of past infection cannot be overstated. A test that could identify the 10% who are infected should reduce the cost and morbidity of treatment 10-fold while maintaining the effectiveness of the intervention. Until we have such a test, our analysis does not affect clinical guidelines for the current methods of detection and preventive treatment of TB infection; rather, our analysis reinforces that a key aspect of TB prevention strategies is to target the people at highest risk, who stand to benefit the most from intervention.

Finally, if 90% of people with TB immunoreactivity do not develop active TB when challenged with TNF neutralising antibodies, HIV, anti-rejection drugs or haematopoietic stem cell transplantation, it is unlikely that a central assumption in the global burden of TB infection has been fulfilled. Current estimates indicate that 25-30% of the world’s population are infected with TB and at risk of TB disease based on the explicit premise that infection is life long.^30^ If people are capable of clearing infection, then estimates of the global prevalence of infection, which are based on the cumulative incidence of new infections, need to be revisited and revised.

## Web extra

Extra material supplied by authors

**Supplementary table 1:** Types of immunosuppression

**Supplementary methods**

**Supplementary table 2:** BCG vaccination policies and possible effect of BCG vaccine on TB prevalence estimates

## Notes

Contributors and sources: The contributors are clinician-scientists with a longstanding interest in the pathogenesis and epidemiology of TB. MAB is an expert in bacterial genomics and molecular epidemiology of TB. PHE is an expert in clinical microbiology and infectious diseases. LR is an expert in the immunopathogenesis of mycobacterial infections. All authors searched the literature. PHE performed the calculations for the absolute risk of TB in the various cohorts and MB checked the calculations. All authors wrote the paper and designed the figures; PHE prepared the figures. The sources of information used include a contemporary literature search using PubMed and Google Scholar, including searching systematic reviews, a search of older articles from the pre-antibiotic era. PHE is the guarantor.
