# Supplementary material for: Is Mycobacterium tuberculosis infection life long?
Source: BMJ. 2019 Oct 24;367:l5770. doi: 10.1136/bmj.l5770 (PMC6812595; doi:10.1136/bmj.l5770)
Supplement: Supplementary file 1 — Supplementary table 1: Types of immunosuppression [file behm050761.ww1.pdf]

Supplemental Table 1

**Type of immunosuppression**	Active TB (%)	95% CI	N total/TST+ only	TST+ prevalence (%)	TB disease (N)	Data adjusted for:		Observation period
**antiTNF (infliximab)**						IPT[a]	Strain clustering	
US[1]	0.7	0.2-1.8	6460/594	9	4	no	no	1-3 years
US[2]	0.6	0.4-0.9	121,000/2,833	4.2[b]	17[c]	no	no	2 years
France[3]	1.0[d]	0.6-1.8	unknown/1154[e]	22[f]	12[d]	no	no	2-5 years
Spain[4]	4.9	3 to 7.8	1540/347[e]	22[g]	17	no	no	1.1 years
**SOT**								
Turkey kidney[5]	11.3	7.4-17	443/177[e]	40[h]	20	no	no	15 months for 1 grp, 55 months for 2nd grp
US kidney[6]	9.9	7.9-12.4	15,870/666[e]	4.2[b]	66	no	no	1.6 years
Spain multiple organs[7]	4.4	2.9-6.8	4388/471	19	21	yes	no	1 year
**HIV/AIDS (US)**								
US[8]	2.9	0.0-15.8	/35	100[i]	1[j]	no	yes	5 years
US[9]	11.3	6.5-19.0	466/106	25	12	yes	yes	5 years
**HSCT**								
Taiwan[10]	8.0	5.8-10.7	2040/492[e]	24[k]	39	no	no	10 years
US[11]	0	0.0-14.0	29/29	100[i]	0	Yes (none treated)	no	5 years
Korea[12]	10.3	6.4-16.2	550/155[e]	28[l]	16	no (none treated)	no	5.8 years
India[13]	7.1	2.8-16.1	175/70[e]	40[m]	5	no (none treated)	no	3.1 years

Footnotes

a, INH preventive therapy; b, estimated TST positive prevalence based on US average in 1999-2000[14]; c, US patients only, as this was the population for which TB disease rates were determined; d, only patients from 2000 are included because after that INH preventive therapy was given to some or most TST-positive patients; e, estimated number of tuberculin skin test (TST)-positive persons based on number of those with TB and on the % of TST-positive people with active TB; f, estimated TST positive prevalence based on a 2005-2009 French study[15] of patients with chronic inflammatory arthritis who were candidates for biologic therapy, where the T-spot positive prevalence were taken as the more specific figure; g, TST prevalence in a later study of Spanish patients with rheumatologic diseases undergoing skin testing prior to infliximab therapy[16]; h, IGRA-positive prevalence in a study of Turkish patients undergoing testing prior to biologic therapy for inflammatory bowel disease[17]; i, only TST-positive patients were studied; j, of 6 patients with TB, five were clustered, indicating recent infection, not activation of remote TB infection; k, TST+ prevalence is based on the IGRA-positive prevalence of Taiwanese persons[18]; l, IGRA-positive prevalence of HSCT patients in same hospital a few years later[12]; m, TB infection prevalence cited in paper regarding the Indian population, not specifically the study group

References

1. Wolfe F, Michaud K, Anderson J, Urbansky K. Tuberculosis infection in patients with rheumatoid arthritis and the effect of infliximab therapy. Arthritis Rheum 2004;50:372-9.
2. Keane J, Gershon S, Wise RP, et al. Tuberculosis associated with infliximab, a tumor necrosis factor alpha-neutralizing agent. N Engl J Med 2001;345:1098-104.
3. Baldin B, Dozol A, Spreux A, Chichmanian RM. Tuberculoses lors de traitements par l'infliximab. Suivi national du 1er janvier 2000 au 30 juin 2003. Presse Med 2005;34:353-7.
4. Gomez-Reino JJ, Carmona L, Valverde VR, Mola EM, Montero MD, Group B. Treatment of rheumatoid arthritis with tumor necrosis factor inhibitors may predispose to significant increase in tuberculosis risk: a multicenter active-surveillance report. Arthritis Rheum 2003;48:2122-7.
5. Atasever A, Bacakoglu F, Toz H, et al. Tuberculosis in renal transplant recipients on various immunosuppressive regimens. Nephrol Dial Transplant 2005;20:797-802.
6. Klote MM, Agodoa LY, Abbott K. Mycobacterium tuberculosis infection incidence in hospitalized renal transplant patients in the United States, 1998-2000. Am J Transplant 2004;4:1523-8.
7. Torre-Cisneros J, Doblas A, Aguado JM, et al. Tuberculosis after solid-organ transplant: incidence, risk factors, and clinical characteristics in the RESITRA (Spanish Network of Infection in Transplantation) cohort. Clin Infect Dis 2009;48:1657-65.
8. Moss AR, Hahn JA, Tulsky JP, Daley CL, Small PM, Hopewell PC. Tuberculosis in the homeless. A prospective study. Am J Respir Crit Care Med 2000;162:460-4.
9. Horsburgh CR, Jr., O'Donnell M, Chamblee S, et al. Revisiting rates of reactivation tuberculosis: a population-based approach. Am J Respir Crit Care Med 2010;182:420-5.
10. Fan WC, Liu CJ, Hong YC, et al. Long-term risk of tuberculosis in haematopoietic stem cell transplant recipients: a 10-year nationwide study. Int J Tuberc Lung Dis 2015;19:58-64.
11. Cheng MP, Kusztos AE, Bold TD, et al. Risk of Latent Tuberculosis Reactivation after Hematopoietic-Cell Transplantation. Clin Infect Dis 2019:in press.
12. Lee HJ, Lee DG, Choi SM, et al. The demanding attention of tuberculosis in allogeneic hematopoietic stem cell transplantation recipients: High incidence compared with general population. PLoS One 2017;12:e0173250.
13. Agrawal N, Aggarwal M, Kapoor J, et al. Incidence and clinical profile of tuberculosis after allogeneic stem cell transplantation. Transpl Infect Dis 2018;20:e12794.
14. Bennett DE, Courval JM, Onorato I, et al. Prevalence of tuberculosis infection in the United States population: the national health and nutrition examination survey, 1999-2000. Am J Respir Crit Care Med 2008;177:348-55.

15. Costantino F, de Carvalho Bittencourt M, Rat AC, et al. Screening for latent tuberculosis infection in patients with chronic inflammatory arthritis: discrepancies between tuberculin skin test and interferon-gamma release assay results. J Rheumatol 2013;40:1986-93.
16. Gomez-Reino JJ, Carmona L, Angel Descalzo M, Biobadaser G. Risk of tuberculosis in patients treated with tumor necrosis factor antagonists due to incomplete prevention of reactivation of latent infection. Arthritis Rheum 2007;57:756-61.
17. Cekic C, Aslan F, Vatansever S, et al. Latent tuberculosis screening tests and active tuberculosis infection rates in Turkish inflammatory bowel disease patients under anti-tumor necrosis factor therapy. Ann Gastroenterol 2015;28:241-6.
18. Fan WC, Ting WY, Lee MC, et al. Latent TB infection in newly diagnosed lung cancer patients - A multicenter prospective observational study. Lung Cancer 2014;85:472-8.
