# Supplementary material for: Is Mycobacterium tuberculosis infection life long?
Source: BMJ. 2019 Oct 24;367:l5770. doi: 10.1136/bmj.l5770 (PMC6812595; doi:10.1136/bmj.l5770)
Supplement: Supplementary file 2 — Supplementary methods [file behm050761.ww2.pdf]

Literature search

For the absolute risk of TB disease following immune-suppression, we extracted data the primary articles cited in systematic reviews of TB and anti-TNF, and TB and solid organ transplant. For the absolute risk of TB disease in people living with HIV/AIDS, we reviewed the primary data from articles in the N. Menzies 2018 systematic review of TB[1]. For the absolute risk of TB following hematopoietic stem cell transplant, we searched for "tuberculosis" and "HSCT" and "humans" in PubMed and reviewed the references of a 2019 paper on the subject[2], to ensure that we had not overlooked other primary clinical data. For each of these, we estimated the percent of individuals with TST-positivity who did not progress to active TB, based on TST data provided in the primary article. Where the authors did not specify whether TB patients had previously had a positive TST, we made the conservative assumption that all TB cases came from the TST-positive subset. Where TST data was not provided, we estimated the percent of individuals likely to be TST-positive based on studies performed in the same countries. For each study, the percent TST-positive that remained TB free after immune-suppression was estimated from the numbers detailed below and the 95% CIs were generated used the adjusted Wald calculation[3]. For those individuals who did develop active TB, the time course of incident TB cases was used to graphically represent the cumulative risk of disease. Supplemental table 1 summarizes all of these studies. We also determined if BCG vaccination in persons without TB infection could have biased our estimates of TB immunoreactivity prevalence by determining national BCG vaccination policies and the methods used to determine TB immunoreactivity and summarized this in Supplemental table 2.

Absolute Risk of TB Disease – Calculations

1) TNFα inhibition

Effect of TNFα inhibition (TI) on tuberculosis in recipients using studies performed in the era before universal screening for TB infection and INH prophylaxis of infected persons.

USA- Keane[4]

The increased risk of TI for TB became apparent in 2001. This retrospective review of such cases, all treated with infliximab, reported 70 cases occurring a median of 12 weeks after the initiation of infliximab, for arthritis or Crohn's disease. Cases originated in the US, Spain, France, Italy and other European countries, all with low TB incidence rates. The estimated annual incidence rate in US infliximab recipients was 24.4/100,000, above the background rate of 6.2 for the underlying illnesses or the other immunosuppressive drugs given the patients. To calculate the relative risk for TST positive persons receiving infliximab, we used an estimated TST-positivity of 4.2%, based on a 1998-2000 survey[5]. Using a higher TST prevalence, a higher proportion of infliximab treated patients would remain tuberculosis free.

24.4/(100,000*0.042) = 24.4/4200, or 0.0058; or 0.58% of those likely to be TST positive developed active TB. Thus, 99.42% did not develop TB. Keane reported that 17 US persons had TB. 17/X=0.0058, so X= 2931, providing an estimate of 2931 TST positives in that

population. The 95% CI of 17/2931 = 0.0036 to 0.0093. The 95% CI of likely TST positive who remained free of TB is therefore 99.1 to 99.6% and is plotted in Figure 3.

Spain – Gomez-Reino[6]
Of 1578 patients taking infliximab, 17 developed TB. Another report on that cohort[7] stated that 22% were TST-positive. 22% of 1578 = 347. 17/347 = 0.049 developed TB. Conversely, 95.1% did not develop TB. 95% CI = 97.0 to 92.2%.

France - Baldin[8]
This retrospective French study examined French cases in patients receiving infliximab for the period 2000-2003. 100% of participants were taking infliximab. They reported an annual rate of 230/100,000 for cases in 2000, 140/100,000 in 2001 and 80/100,000 in 2002. Because it is unclear whether screening and treatment contributed to the drop in rates over time, we used the largest figure. The TST+ prevalence rate of 22% came from another study[9] of TI-candidates with chronic inflammatory arthritis comparing TST vs Tspot, where the TST rate was 35% and Tspot 22%. We used the lower number (22%) for calculations, recognizing that the higher figure would give a lower absolute rate of TB among TB reactive patients.

230/100,000(.22) = 230/22,000, or 0.0104, 1.04%. 99% likely TST positives remained TB free. Using the same method used for Keane, there were 12 patients with TB in 2000. 12/x=0.0104, X=1154. For 12 cases in 1154 individuals, the 95% CI is 0.0057 to 0.0183; conversely, the 95% CI for remaining TB free was 98.2 to 99.4%.

USA - Wolfe[10]
This American study of a large cohort of patients with inflammatory arthritis taking infliximab in the period 2000-2002, using as a control similar patients not taking TNF-inhibitors (TI) in 1998-1999. 100% of participants were taking infliximab. This found the annual rate to be 54.5/100,000 in the TI phase and 6.2 pre-TI. The TST+ prevalence was given in this study as 9.2%. For this study, there were 6460 patients taking infliximab at a time when no INH prophylaxis was administered. They were followed for two years. 4/6460 (0.06%) of these developed TB; all were elderly women and of these two had known prior +TST and one had a possible history of prior TB (not further defined). 9.2% of the 1998-1999 study group of arthritis patients (pre-infliximab) were TST+. Assuming that all patients who developed TB came from the TST+ population (9.2% of 6460, or 594), 4/594 was the TB disease fraction, or 0.67%. 95% CI of 4/594 is 0.2 to 1.8%. Thus 99.33% of the TST+ population did not develop TB over a two-year period of infliximab (95% CI: 98.2 to 99.8%).

2) HIV

San Francisco, USA[11]

40 HIV+ TST+ homeless individuals. 6 developed TB. By RFLP, 5/6 were in clusters and assigned by the authors to recent infection. Using the data without respect to RFLP, 34/40 = 85%, 70.4 to 93.2%. An adjusted analysis excluded the 5 who developed TB as part of an RFLP-

defined cluster. Of the remaining 35 individuals, 1 developed TB. Therefore, 34/35 TST+ HIV+ homeless individuals remained TB-free (97.1%, 95% CI: 83.9 to 100), over 5 years. INH prophylaxis was incomplete - none completed even a five month course. No adjustment was made for IPT.

Florida, USA[12]

Using RFLP as the genotyping method, 27 non-clustered cases of TB were identified. 15 were in HIV-negative individuals. The 12 non-clustered TB cases came from 106 TST+ HIV+ patients who developed TB and who had not received INH preventive therapy. Most were moderately immunosuppressed (CD4 ~200). The TST+ prevalence for the HIV-positive population was 25% (all from Table 1). TB-free percent was 94/106 = 88.7 (95% CI: 81 to 93.5).

3) Solid organ transplantation

Turkey - kidney transplant recipients[13]

Atasever et al report on renal transplant recipients in Turkey. 443 transplants were studied, of whom 20 developed TB post-transplant. One of the 20 had TB 18 years earlier and four had CXR findings c/w old TB. Based on several studies, the TST+ prevalence in Turkey is around 40 to 70%; we used the 40% figure as it has been described in IGRA surveys[14] and is likely is a better approximation for this BCG-vaccinated population. 40% of 443 is 177. 20/177 is the fraction of likely TST+ patients with TB, or 11.3%. 89% of these transplant patients who were likely TST+ did not develop TB. 95% CI is 83 to 92.6

USA - kidney transplant recipients[15]

1998-2000 study of US Medicare recipients having a renal transplant. 66 of 15,870 developed TB, or 0.42%. The US TST prevalence in 1999-2000 was estimated to be 4.2%[5]. 4.2% of 15,870 is 666. 66/666 = 9.90%. 100-9.9 = 90.1% were TB free. 95% CI 87.6 to 92.1

Spain - solid organ transplant recipients[16]

From September 2003 to June 2006, 4388 solid organ transplants were done.

Organ	TB/total	TB%	TST + all patients (N)	%TST+ received INH
heart	1/404	0.25	15.0	75
kidney	7/2052	0.34	13.6	52.9
liver	8/1507	0.53	23.3	29.6
kidney-pancreas	1/122	0.82	23.5	50
lung	4/303	1.32	27.3	46.8
total	21/4388	0.48	19.0	43.5

Assuming that all who received INH prophylaxis were not at risk, 56.5% remained at risk. 19% of 4388 = 834 TST+. 56.5% of 834 = 471 (TST+ not receiving INH). 21/471 = 4.5% of likely TST positive not receiving INH prophylaxis. Note that 3 of the 21 who developed TB were TST negative, so the denominator of those at risk is likely higher and therefore a higher percentage remained TB free. However, assuming that all who developed TB were from the TST positive group not receiving INH, 95.5% remained TB free (95%: CI: 95% CI 93.2 to 97.1)

4) HSCT

Taiwan Fan 2015[17],

Stem cell transplants Taiwan. The total number of TB cases in 2040 recipients was 39, or 1.91%. Observation period 10 years. Using a study of controls from a study of Taiwanese with lung cancer [18], the IGRA+ rate in Taiwan was 23.8% (rounded to 24%). The TST+ population size is then estimated at 0.24 x 2040, or 490 patients. 39/490 = 8.0%. TB free is 92.0%, 95% CI = 89.3 to 94.2%

USA - Cheng 2019[2],

0 of 29 TST + HSCT patients developed TB over a five year period. Data are given as person years, but based on this it seems that observation was around 5 years or longer. 95% CI of 29/29 = 86.1 to 100.

South Korea - Lee 2017[19]

16 of 550 HSCT patients overall developed TB during a time when TST/IGRA were not being used to screen HSCT candidates. The IGRA positivity rate at the same hospital for HSCT candidates a few years later was 28.1% (mid-2009 to 2011 vs. 2004-early 2009 when no IGRA tests done). 28% of 550 = 155 with TST+. 16/155 = 10.3% (95% CI 6.4 to 16.2). TB calculated as 89.7% (95% CI: 83.8 to 93.6%).

India - Agrawal,2018[20]

No TST/IGRA done and no IPT administered. 5 of 175 transplants developed TB (2.9%). Paper says TBI prevalence is 40% in India. 40% of 175 = 70. 5/70 = 7.1% (2.8 to 16.1%). TB free is 92.8% (83.9 to 97.2% 95% CI)

1. Menzies NA, Wolf E, Connors D, et al. Progression from latent infection to active disease in dynamic tuberculosis transmission models: a systematic review of the validity of modelling assumptions. Lancet Infect Dis 2018;18:e228-e38.

2. Cheng MP, Kusztos AE, Bold TD, et al. Risk of Latent Tuberculosis Reactivation after Hematopoietic-Cell Transplantation. Clin Infect Dis 2019:in press.
3. Agresti A, Coull BA. Approximate is Better than "Exact" for Interval Estimation of Binomial Proportions. The American Statistician 1998;52:119-26.
4. Keane J, Gershon S, Wise RP, et al. Tuberculosis associated with infliximab, a tumor necrosis factor alpha-neutralizing agent. N Engl J Med 2001;345:1098-104.
5. Bennett DE, Courval JM, Onorato I, et al. Prevalence of tuberculosis infection in the United States population: the national health and nutrition examination survey, 1999-2000. Am J Respir Crit Care Med 2008;177:348-55.
6. Gomez-Reino JJ, Carmona L, Valverde VR, Mola EM, Montero MD, Group B. Treatment of rheumatoid arthritis with tumor necrosis factor inhibitors may predispose to significant increase in tuberculosis risk: a multicenter active-surveillance report. Arthritis Rheum 2003;48:2122-7.
7. Gomez-Reino JJ, Carmona L, Angel Descalzo M, Biobadaser G. Risk of tuberculosis in patients treated with tumor necrosis factor antagonists due to incomplete prevention of reactivation of latent infection. Arthritis Rheum 2007;57:756-61.
8. Baldin B, Dozol A, Spreux A, Chichmanian RM. Tuberculoses lors de traitements par l'infliximab. Suivi national du 1er janvier 2000 au 30 juin 2003. Presse Med 2005;34:353-7.
9. Costantino F, de Carvalho Bittencourt M, Rat AC, et al. Screening for latent tuberculosis infection in patients with chronic inflammatory arthritis: discrepancies between tuberculin skin test and interferon-gamma release assay results. J Rheumatol 2013;40:1986-93.
10. Wolfe F, Michaud K, Anderson J, Urbansky K. Tuberculosis infection in patients with rheumatoid arthritis and the effect of infliximab therapy. Arthritis Rheum 2004;50:372-9.
11. Moss AR, Hahn JA, Tulsky JP, Daley CL, Small PM, Hopewell PC. Tuberculosis in the homeless. A prospective study. Am J Respir Crit Care Med 2000;162:460-4.
12. Horsburgh CR, Jr., O'Donnell M, Chamblee S, et al. Revisiting rates of reactivation tuberculosis: a population-based approach. Am J Respir Crit Care Med 2010;182:420-5.
13. Atasever A, Bacakoglu F, Toz H, et al. Tuberculosis in renal transplant recipients on various immunosuppressive regimens. Nephrol Dial Transplant 2005;20:797-802.
14. Cekic C, Aslan F, Vatansever S, et al. Latent tuberculosis screening tests and active tuberculosis infection rates in Turkish inflammatory bowel disease patients under anti-tumor necrosis factor therapy. Ann Gastroenterol 2015;28:241-6.
15. Klote MM, Agodoa LY, Abbott K. Mycobacterium tuberculosis infection incidence in hospitalized renal transplant patients in the United States, 1998-2000. Am J Transplant 2004;4:1523-8.
16. Torre-Cisneros J, Doblas A, Aguado JM, et al. Tuberculosis after solid-organ transplant: incidence, risk factors, and clinical characteristics in the RESITRA (Spanish Network of Infection in Transplantation) cohort. Clin Infect Dis 2009;48:1657-65.
17. Fan WC, Liu CJ, Hong YC, et al. Long-term risk of tuberculosis in haematopoietic stem cell transplant recipients: a 10-year nationwide study. Int J Tuberc Lung Dis 2015;19:58-64.
18. Fan WC, Ting WY, Lee MC, et al. Latent TB infection in newly diagnosed lung cancer patients - A multicenter prospective observational study. Lung Cancer 2014;85:472-8.
19. Lee HJ, Lee DG, Choi SM, et al. The demanding attention of tuberculosis in allogeneic hematopoietic stem cell transplantation recipients: High incidence compared with general population. PLoS One 2017;12:e0173250.
20. Agrawal N, Aggarwal M, Kapoor J, et al. Incidence and clinical profile of tuberculosis after allogeneic stem cell transplantation. Transpl Infect Dis 2018;20:e12794.
