# Supplementary material for: Is Mycobacterium tuberculosis infection life long?
Source: BMJ. 2019 Oct 24;367:l5770. doi: 10.1136/bmj.l5770 (PMC6812595; doi:10.1136/bmj.l5770)
Supplement: Supplementary file 3 — Supplementary table 2: BCG vaccination policies and possible effect of BCG vaccine on TB prevalence estimates [file behm050761.ww3.pdf]

Supplemental Table 2

BCG vaccination policies[a] and possible effect of BCG vaccine on TB prevalence estimates

**Type of immunosuppression**	BCG vaccine used?	BCG vaccination stopped	BCG given <1 yr	BCG boosters >1 yr	Tuberculin reactivity assay positive with BCG boosters >1 yr[1]	Effect of BCG vaccination on TB prevalence estimate
**antiTNF**						
US[2]	no	NA	NA	NA	no (no BCG)	none
US[3]	no	NA	NA	NA	no (no BCG)	none
France[4]	yes	2007	yes	no	no (Tspot)[5, b]	none
Spain[6]	yes	1981	yes	no	no (no boosters used)	none
**SOT**						
Turkey kidney[7]	yes	no	yes	until 2006	no (IGRA)[5, c]	none
US kidney[8]	no	NA	NA	NA	no (no BCG)	none
Spain multiple organs[9]	yes	1981	yes	no	no (no boosters used)	none
**HIV/AIDS (US)**						
US[10]	no (90% US born	NA	NA	NA	no (no BCG)	none
US[11]	yes – Haitians only data	no (Haiti)	yes (Haiti)	no (Haiti)	no (no boosters used in Haiti)	none
**HSCT**						
Taiwan[12]	yes	no	yes	until 1997	no (IGRA)[d]	none

US[13]	no	NA	NA	NA	no (no BCG)	none
Korea[14]	yes	no	yes	until 2007	no (IGRA)[e]	none
India[15]	yes	no	yes	no	no (no boosters used)	none

Footnotes

a, BCG vaccination policies were obtained from the BCG World Atlas (http://bcgatlas.org/index.php); b, estimated TST positive prevalence based on a 2005-2009 French study[16] of patients with chronic inflammatory arthritis who were candidates for biologic therapy, where the T-spot positive prevalence were taken as the more specific figure; c, IGRA-positive prevalence in a study of Turkish patients undergoing testing prior to biologic therapy for inflammatory bowel disease[17]; d, TST+ prevalence is based on the IGRA-positive prevalence of Taiwanese persons[18]; e, IGRA-positive prevalence of HSCT patients in same hospital a few years later[14]

References

1. Farhat M, Greenaway C, Pai M, Menzies D. False-positive tuberculin skin tests: what is the absolute effect of BCG and non-tuberculous mycobacteria? Int J Tuberc Lung Dis 2006;10:1192-204.
2. Wolfe F, Michaud K, Anderson J, Urbansky K. Tuberculosis infection in patients with rheumatoid arthritis and the effect of infliximab therapy. Arthritis Rheum 2004;50:372-9.
3. Keane J, Gershon S, Wise RP, et al. Tuberculosis associated with infliximab, a tumor necrosis factor alpha-neutralizing agent. N Engl J Med 2001;345:1098-104.
4. Baldin B, Dozol A, Spreux A, Chichmanian RM. Tuberculoses lors de traitements par l’infliximab. Suivi national du 1er janvier 2000 au 30 juin 2003. Presse Med 2005;34:353-7.
5. Pai M, Denkinger CM, Kik SV, et al. Gamma interferon release assays for detection of Mycobacterium tuberculosis infection. Clin Microbiol Rev 2014;27:3-20.
6. Gomez-Reino JJ, Carmona L, Valverde VR, Mola EM, Montero MD, Group B. Treatment of rheumatoid arthritis with tumor necrosis factor inhibitors may predispose to significant increase in tuberculosis risk: a multicenter active-surveillance report. Arthritis Rheum 2003;48:2122-7.
7. Atasever A, Bacakoglu F, Toz H, et al. Tuberculosis in renal transplant recipients on various immunosuppressive regimens. Nephrol Dial Transplant 2005;20:797-802.
8. Klote MM, Agodoa LY, Abbott K. Mycobacterium tuberculosis infection incidence in hospitalized renal transplant patients in the United States, 1998-2000. Am J Transplant 2004;4:1523-8.

9. Torre-Cisneros J, Doblas A, Aguado JM, et al. Tuberculosis after solid-organ transplant: incidence, risk factors, and clinical characteristics in the RESITRA (Spanish Network of Infection in Transplantation) cohort. Clin Infect Dis 2009;48:1657-65.
10. Moss AR, Hahn JA, Tulsky JP, Daley CL, Small PM, Hopewell PC. Tuberculosis in the homeless. A prospective study. Am J Respir Crit Care Med 2000;162:460-4.
11. Horsburgh CR, Jr., O'Donnell M, Chamblee S, et al. Revisiting rates of reactivation tuberculosis: a population-based approach. Am J Respir Crit Care Med 2010;182:420-5.
12. Fan WC, Liu CJ, Hong YC, et al. Long-term risk of tuberculosis in haematopoietic stem cell transplant recipients: a 10-year nationwide study. Int J Tuberc Lung Dis 2015;19:58-64.
13. Cheng MP, Kusztos AE, Bold TD, et al. Risk of Latent Tuberculosis Reactivation after Hematopoietic-Cell Transplantation. Clin Infect Dis 2019:in press.
14. Lee HJ, Lee DG, Choi SM, et al. The demanding attention of tuberculosis in allogeneic hematopoietic stem cell transplantation recipients: High incidence compared with general population. PLoS One 2017;12:e0173250.
15. Agrawal N, Aggarwal M, Kapoor J, et al. Incidence and clinical profile of tuberculosis after allogeneic stem cell transplantation. Transpl Infect Dis 2018;20:e12794.
16. Costantino F, de Carvalho Bittencourt M, Rat AC, et al. Screening for latent tuberculosis infection in patients with chronic inflammatory arthritis: discrepancies between tuberculin skin test and interferon-gamma release assay results. J Rheumatol 2013;40:1986-93.
17. Cekic C, Aslan F, Vatansever S, et al. Latent tuberculosis screening tests and active tuberculosis infection rates in Turkish inflammatory bowel disease patients under anti-tumor necrosis factor therapy. Ann Gastroenterol 2015;28:241-6.
18. Fan WC, Ting WY, Lee MC, et al. Latent TB infection in newly diagnosed lung cancer patients - A multicenter prospective observational study. Lung Cancer 2014;85:472-8.
